# An experimental study on lung deposition of inhaled 2 μm particles in relation to lung characteristics and deposition models

**DOI:** 10.1186/s12989-023-00551-9

**Published:** 2023-10-24

**Authors:** Jenny Rissler, Madeleine Peterson Sjögren, Julia Linell, Amalia Larsson Hurtig, Per Wollmer, Jakob Löndahl

**Affiliations:** 1 Ergonomics and Aerosol Technology, Lund University, Lund, 22100 Sweden; 2 NanoLund, Lund University, Lund, 22100 Sweden; 3 RISE Research Institutes of Sweden, Lund, 22370 Sweden; 4 Department of Translational Medicine, Clinical Physiology and Nuclear Medicine, Lund University, Malmö, 20502 Sweden

**Keywords:** Lung deposition, Respiratory tract, Particle deposition, AiDA, Health effects of aerosols, Inhaled dose, Pulmonary

## Abstract

**Background:**

The understanding of inhaled particle respiratory tract deposition is a key link to understand the health effects of particles or the efficiency for medical drug delivery via the lung. However, there are few experimental data on particle respiratory tract deposition, and the existing data deviates considerably when comparing results for particles > 1 μm.

**Methods:**

We designed an experimental set-up to measure deposition in the respiratory tract for particles > 1 μm, more specifically 2.3 μm, with careful consideration to minimise foreseen errors. We measured the deposition in seventeen healthy adults (21–68 years). The measurements were performed at tidal breathing, during three consecutive 5-minute periods while logging breathing patterns. Pulmonary function tests were performed, including the new airspace dimension assessment (AiDA) method measuring distal lung airspace radius (*r*_AiDA_). The lung characteristics and breathing variables were used in statistical models to investigate to what extent they can explain individual variations in measured deposited particle fraction. The measured particle deposition was compared to values predicted with whole lung models. Model calculations were made for each subject using measured variables as input (e.g., breathing pattern and functional residual capacity).

**Results:**

The measured fractional deposition for 2.3 μm particles was 0.60 ± 0.14, which is significantly higher than predicted by any of the models tested, ranging from 0.37 ± 0.08 to 0.53 ± 0.09. The multiple-path particle dosimetry (MPPD) model most closely predicted the measured deposition when using the new PNNL lung model. The individual variability in measured particle deposition was best explained by breathing pattern and distal airspace radius (*r*_AiDA_) at half inflation from AiDA. All models underestimated inter-subject variability even though the individual breathing pattern and functional residual capacity for each participant was used in the model.

**Conclusions:**

Whole lung models need to be tuned and improved to predict the respiratory tract particle deposition of micron-sized particles, and to capture individual variations – a variation that is known to be higher for aged and diseased lungs. Further, the results support the hypothesis that the AiDA method measures dimensions in the peripheral lung and that *r*_AiDA_, as measured by the AiDA, can be used to better understand the individual variation in the dose to healthy and diseased lungs.

**Supplementary Information:**

The online version contains supplementary material available at 10.1186/s12989-023-00551-9.

## Introduction

Knowledge of the respiratory tract deposition of inhaled particles is a key link to understand the health effect of air pollution, transmission of airborne diseases, and the efficiency for medical drug delivery via the lung. Respiratory tract deposition of aerosols is, however, complex, and there are considerable deviations between measured and modelled data on respiratory tract deposition and between the experimental results reported [1–5].

A major reason for the variation in the measured respiratory tract deposited fraction (DF) is likely experimental limitations, and the fact that there is no standard methodology for measuring DF of inhaled particles. For example, many studies have overlooked critical methodological aspects that may have biased data [6]. Additionally, often essential information about lung function, breathing pattern and other subject characteristics are not reported, and most studies use relatively small groups of volunteers – typically less than 10, whereof a majority have been men. This also explain part of the variation in the reported DFs. Notably, this is the case for some of the most important works, such as those by Heyder et al. [7, 8] and Schiller et al. [9, 10], that laid the foundation for later models of respiratory tract deposition.

Model calculations of respiratory tract particle deposition are also uncertain due to simplifications regarding lung geometry and airflows, and due to computational constraints. Especially for the deep lung, it is difficult to model the DF of aerosol particles due to this region’s complex network of airspaces with irregular geometrical structures that vary in size during the breathing cycle. This part of the lung is also less well known from physiological examinations since it is difficult to measure peripheral pulmonary function and structure with standard pulmonary function tests (PFTs) [11, 12]. Histology may be an alternative, but it is not fully representative for the healthy population and difficult to relate to other lung function data. Nonetheless, the alveolar region constitutes more than 90% of the lungs by volume and is hence critical for understanding of the DF of inhaled aerosols.

In a previous study, including ~ 70 subjects in a wide age span (from ~ 7 to 70 years), we noted that for particles with diameters in the range 1–5 μm, the measured DF (DF_meas_) were systematically higher compared to those modelled using the National Council on Radiation Protection and Measurement (NCRP) and International Commission on Radiological Protection (ICRP) models [1]. The earlier experimental data on respiratory particle deposition reported in the literature for particles in this size range varies considerably. For instance, DF measured for 1 μm particles during normal breathing vary from 0.1 to 0.7 between studies [e.g. 1, 2, 8, 13, 14, 15, 16, 17, 18, 19]. None of these studies provide any information about the physiology of the alveolar region of the lungs. The main reason for this is that such measurements have been inaccessible.

The aim of this work is to determine the deposition of supermicrometer particles in the respiratory tract and investigate the factors that govern the inter-subject variability in deposition. Moreover, we want to investigate if the findings in our earlier study can be reproduced, where DF_meas_ was systematically higher than that predicted by whole lung models [1]. A a new set-up for respiratory tract deposition measurements was designed and constructed, optimised regarding precision and accuracy. To minimize errors, monodispersed particles were used. For the purpose, particles with a diameter of ~ 2 μm were selected since, during tidal breathing, particles of this size mainly deposit in the peripheral lung [1, 2, 20] – a sensitive part of the respiratory tract. The lung function assessments included the recently developed AiDA method, which provides information about the size of the airspaces in the acinar region of the lungs [21, 22]. The DF_meas_ was compared to that modelled by the most common whole lung models: semi-empirical regional compartment models (ICRP and NCRP), a multiple path model (MPPD), and to a parametrization described by Kim and Hu [17].

## Results

**Fig. 1:**
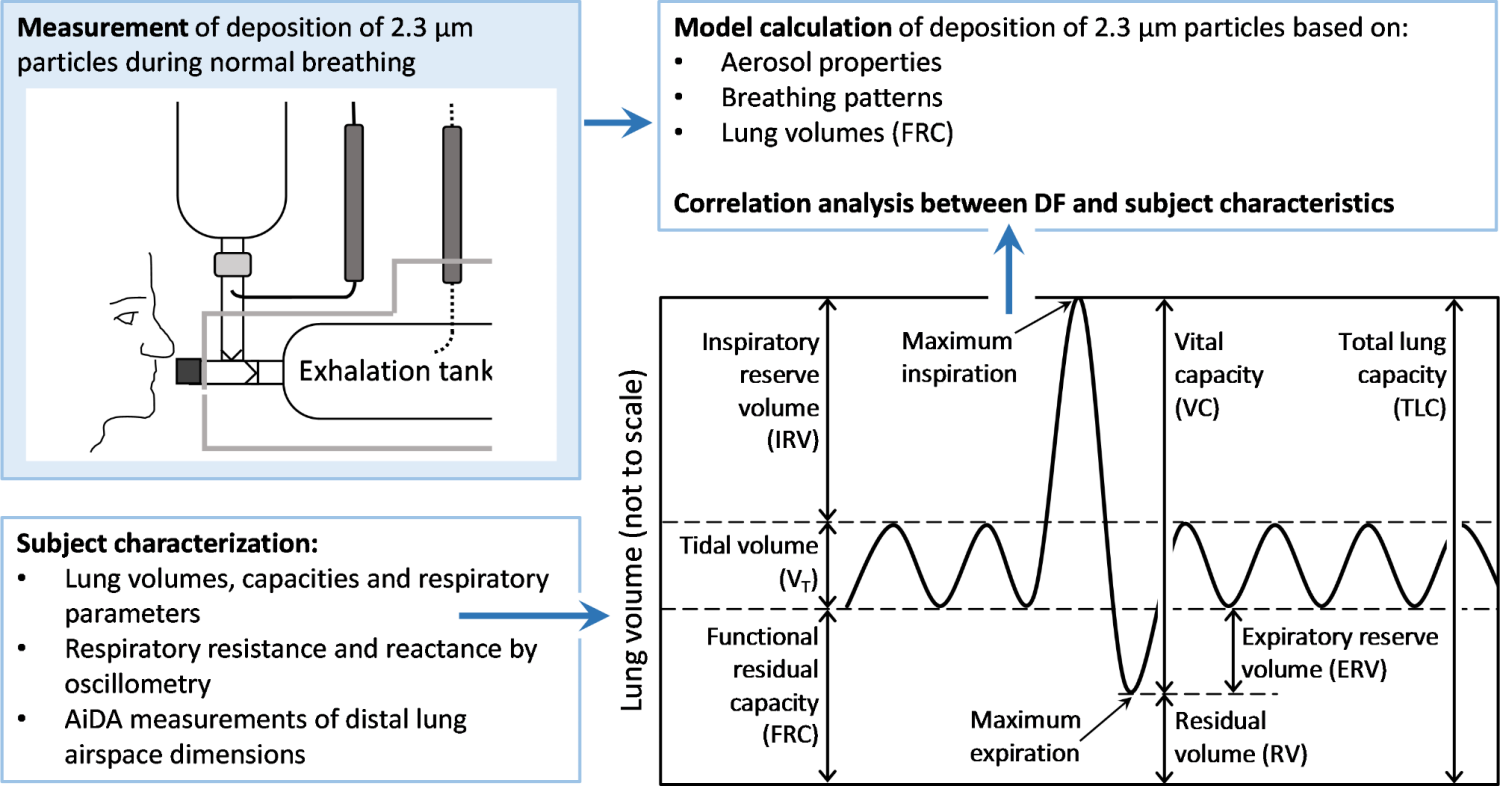
Study overview and graphical definition of lung function parameters

An overview of the study, including a graphical definition of the main lung function parameters, is provided in Fig. 1.

### Lung function data, lung characteristics and deposited particle fraction

The average DF_meas_ for 2.3 μm particles was experimentally determined to 0.60 ± 0.12 (mean ± 1 SD), measured under tidal breathing (inhalation and exhalation during restful breathing) in 17 healthy subjects, aged 21–68 years. Lung function data, respiratory parameters, additional measured lung characteristics, and background variables are summarized in Table 1, together with DF_meas_. The corresponding data for all individuals are presented in Additional file 1, Table A1. Each measurement session was divided into three 5-minute periods. The average respiratory parameters (tidal volume, *V*_*T*_, and breathing cycle time, *T*_*bc*_) during the three periods were compared to see if there were any trends explained, for example, by that the subjects got more relaxed with time. A significant decrease in *V*_*T*_ (p = 0.003) was observed over time, mainly occurring between period 1 and 2. Breathing cycle time, *T*_*bc*_, increased between period 1 and 3, although not significant. This led to an overall decrease in the minute volume ventilation rate, *V*_e_, with time, most prominant between period 1 and period 3 (p = 0.001).

**Table 1:** Overview of recruited subjects including background variables, respiratory parameters, lung function, and DF_meas_, measured at rest

Characteristic	All	Women	Men
Age (years)	47 ± 14	43 ± 15	51 ± 12
Subjects	17	8	9
Height (cm)	179 ± 8	172 $\pm$4	184 ± 7
Weight (kg)	77 ± 13	70 ± 5	85 ± 14
*V*_T_ (L)	0.94 ± 0.34	0.78 ± 0.22	1.09 ± 0.37
*T*_bc_ (min)	0.109 ± 0.045	0.093 ± 0.029	0.124 ± 0.053
*V*_e_ (L/min)	8.8 ± 0.9	8.5 ± 0.7	9.0 ± 1.0
DF_meas_ (a.u.)	0.60 ± 0.12	0.53 ± 0.19	0.64 ± 0.13
*r*_AiDA_ (µm)	280 ± 36	260 ± 34	293 ± 32
*R*_0_ (a.u)	0.59 ± 0.16	0.53 ± 0.19	0.64 ± 0.13
VC_AiDA_ (L)	4.3 ± 0.6	3.9 ± 0.5	4.5 ± 0.6
*r*_AiDA,1/2_ (µm)	261 ± 36	250 ± 23	268 ± 35
*R*_0,1/2_ (a.u.)	0.44 ± 0.14	0.34 ± 0.13	0.50 ± 0.11
½VC_AiDA_ (L)	2.5 ± 0.4	2.3 ± 0.3	2.8 ± 0.4
R_5_ (kPa s/L)	0.33 ± 0.08	0.39 ± 0.07	0.30 ± 0.06
R_20_ (kPa s/L)	0.32 ± 0.08	0.38 ± 0.07	0.28 ± 0.06
TLC (L)	7.3 ± 1.1	6.3 ± 0.6	8.0 ± 0.7
FRC (L)	3.9 ± 0.9	3.4 ± 0.7	4.2 ± 0.9
FEV_1_ (L)	3.8 ± 0.8	3.4 ± 0.3	4.2 ± 0.9
VC (L)	5.0 ± 0.9	4.3 ± 0.3	5.6 ± 0.7
RV (L)	2.2 ± 0.5	1.9 ± 0.4	2.4 ± 0.6
D_LCO_ (mmol min^− 1^ kPa^− 1^)	9.2 ± 2.0	7.3 ± 0.3	10.5 ± 1.6
FEV_1_/VC	0.764 ± 0.081	0.788 ± 0.078	0.742 ± 0.081
TLC (of pred.)	1.07 ± 0.09	1.09 ± 0.11	1.05 ± 0.07
FRC (of pred.)	1.14 ± 0.23	1.14 ± 0.23	1.14 ± 0.24
FEV_1_ of pred.)	1.08 ± 0.12	1.11 ± 0.14	1.05 ± 0.11
VC (of pred.)	1.16 ± 0.14	1.21 ± 0.19	1.13 ± 0.08
RV (of pred.)	1.02 ± 0.15	1.01 ± 0.12	1.03 ± 0.17
FEV_1_/VC (of pred.)	0.97 ± 0.08	0.97 ± 0.07	0.95 ± 0.09

Averages ± 1 SD. Abbreviations: *V*_T_ tidal volume; *T*_bc_ time of breath cycle; *V*_e_ minute volume ventilation rate; DF_meas_ measured total deposited fraction; TLC total lung capacity; FRC functional residual capacity; FEV_1_ forced expiratory volume in 1 s; RV residual volume; VC vital capacity; *R*_0_ (and *R*_0,1/2_) and *r*_AiDA_ (and *r*_AiDA,1/2_) zero seconds recovery and airspace size derived from AiDA measurements at VC_AiDA_ (and ½ VC_AiDA_), respectively; R_5_ and R_20_ respiratory resistance at 5 and 20 Hz from oscillometry, respectively. Predicted values are calculated according to Quanjer et al. [23].

The lung function examination included airspace dimension assessment (AiDA) technique. AiDA is a new method where the average radii of distal airspaces (*r*_AiDA_), primarily in the acinar region of the lungs, are measured at full lung inflation. The mean *r*_AiDA_ for the group was 280 ± 36 μm, with slightly smaller radius for women compared to men. In this study, *r*_AiDA_ was for the first time also measured after inhalation of half vital capacity (denoted *r*_AiDA,1/2_), as this presumably would provide peripheral airspace dimensions closer to those during normal tidal breathing (compare average lung volume during tidal breathing of 4.37 L (FRC + ½ *V*_T_) and the lung volume for AiDA measured after inhalation of half vital capacity of 4.43 L (RV + ½VC_AiDA_). The measured *r*_AiDA,1/2_ was significantly lower than *r*_AiDA_ (p = 0.007), with *r*_AiDA,1/2_ = 261 ± 36 μm (paired t-test). The ratio between *r*_AiDA_ at full inflation volume (VC_AiDA_) and *r*_AiDA,1/2_ at half inflation (½VC_AiDA_) was 1.11. This should be compared to the expected ratio between *r*_AiDA_ and *r*_AiDA,1/2_ of 1.09 if assuming a symmetrical expansion of the respiratory tract at the current inhaled volumes (see Eq. 3).

### Correlation analysis DF and lung characteristics

The results from the Pearson correlation analysis between lung function indices and DF_meas_ are presented in Table 2. The DF_meas_ was linearly correlated with lung function indices VC and FEV_1_, and with measured AiDA index *R*_0_ and *r*_AiDA,1/2_ (Table 2). The AiDA index *R*_0_ (“zero seconds recovery”) is assumed to be related to particle deposition in the small conducting airways during breathing [22] but has not yet been fully evaluated. The DF_meas_ also showed a significant correlation with respiratory parameters *V*_T_ and *T*_bc_. The correlation between ln(*V*_T_) and ln(*T*_bc_) with DF_meas_ (as derived by Kim and Hu [17]) was also evaluated showing that the correlation was stronger for ln(*V*_T_) and ln(*T*_bc_) compared to *V*_T_ and *T*_bc_.

Multivariate linear regression analysis was performed to further investigate which lung indices best predicted the inter-subject variability in DF_meas_. For this, VC, *R*_0_, *r*_AiDA,1/2_, ln(*T*_bc_) and ln(*V*_T_) were used as input parameters to predict DF_meas_. The selection of parameters was based on the Pearson correlation (Table 2). FEV_1_ and *r*_AiDA_ were excluded from the regression analysis since they are strongly correlated with VC and *r*_AiDA,1/2_, respectively, and as their linear correlation with DF was weaker than VC and *r*_AiDA,1/2_. By the same reason ln(*T*_bc_) and ln(*V*_T_) was selected prior to *T*_bc_ and *V*_T_. Relationships between all lung function indices and background variables, assessed with Pearson correlations, is presented in Table A2, Additional file 2. The results of the Multiple-linear regression for DF_meas_ are presented in Table A1 in Additional file 2. The strongest correlations were found for r_AiDA,1/2_ and ln(*T*_bc_). Replacing *r*_AiDA_ with *r*_AiDA,½_ in the model did not result in any significant correlation.

Table A2 suggest that TLC, *V*_T_, FRC, FEV_1_/VC, X_5_, K_CO_, ½VC_AiDA_, R_0_, *r*_AiDA_ and *r*_AiDA,1/2_ are related to age. These correlations are expected.

**Table 2:** Pearson correlation coefficient (*r*) for total deposited fraction (DF) and lung characteristics

Characteristic	Pearson’s correlation coefficient *r*	p-value
Age	0.03	0.9
Height	0.49	0.05
Weight	0.50	0.04
*V*_T_	0.83	< 0.0001
ln(*V*_T_)	0.87	< 0.0001
*T*_bc_	0.80	< 0.0001
ln(*T*_bc_)	0.87	< 0.0001
*V*_e_	0.83	< 0.0001
*r*_AiDA_	-0.13	0.6
*R*_0_	-0.58	0.02
*r*_AiDA,1/2_	-0.58	0.02
*R*_0,1/2_	-0.03	0.9
R_5_	0.24	0.4
R_20_	0.09	0.7
TLC	0.29	0.3
FRC	-0.34	0.2
FEV_1_	0.63	0.006
VC	0.56	0.02
RV	-0.35	0.2
D_LCO_	0.52	0.05

### Comparison and correlation analysis between measured and modelled DF

The DF_meas_ was compared to values modelled by whole lung models (DF_mod_). The ICRP model [24], NCRP model [25] and a multiple path model (MPPD©, Applied Research Associates, Inc., Albuquerque, NM, USA) [26, 27] were used with *V*_T_, *T*_bc_ (logged during the deposition measurements), and FRC of each subject as model input. MPPD was applied with two different lung geometries: Yeh and Schum [28] and PNNL [29], here denoted MPPD-Y&S and MPPD-PNNL, respectively. The models where FRC is used for scaling of the lungs are hereafter collectively referred to as “Group A”. The DF_mod_ was also calculated using a simplified empirical model accounting for variations in the breathing pattern (*V*_*T*_ and *T*_*bc*_) but not any scaling of the lung [17], referred to as K&H. The models NCRP and MPPD-PNNL, were also run keeping FRC fixed to 3.9 L, representing the group average FRC, indicated by subscript F. Together K&H, NCRP_F_ and MPPD-PNNL_F_, are hereafter collectively referred to as “Group B”.

**Fig. 2:**
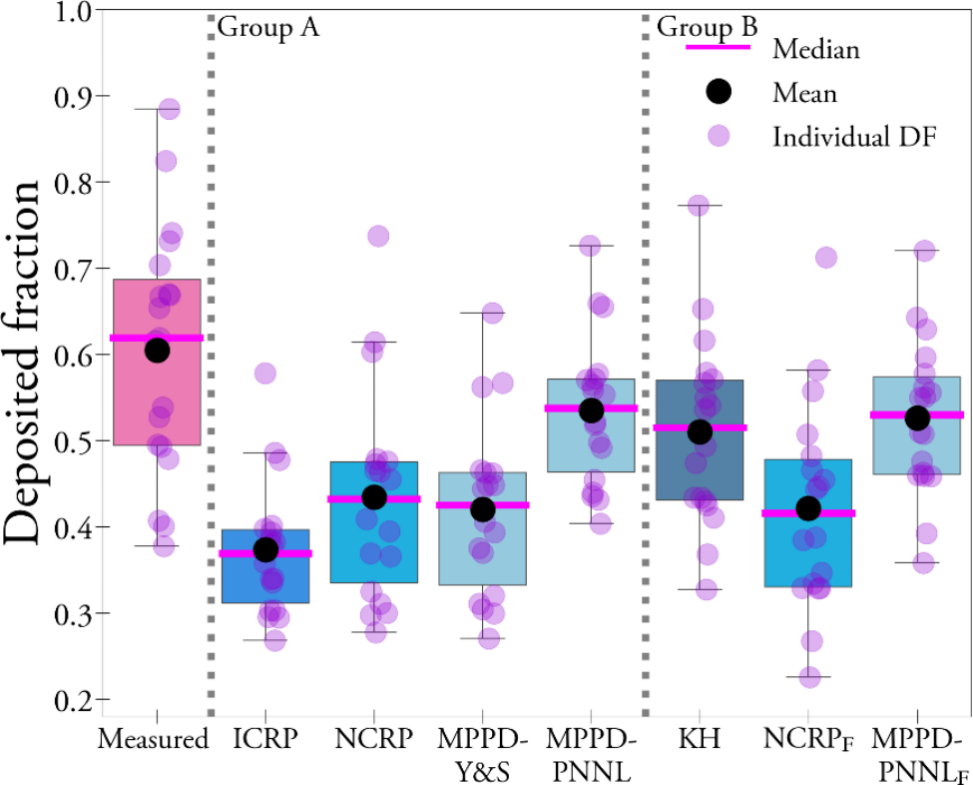
Measured and modelled DF, for models ICRP, NCRP, MPPD, and K&H. Subscript F denotes the modelled DF when keeping FRC at a fixed value, in this case 3.9 L (average of the group). Individual values are shown as circles. Lower and upper limits of each box represent 25th and 75th percentiles, respectively. Vertical bars at the end of lower and upper vertical bars represent 5th and 95th percentiles, respectively

All models predicted significantly lower DF_mod_ compared to DF_meas_, according to analysis of variance and paired t-tests (Table 3). Figure 2 shows the resulting DF_meas_ and DF_mod_ for each model as a boxplot. The model that best predicted average DF_meas_ was the MPPD-PNNL, followed by the K&H parametrization. The average DF_mod_ was lowest for NCRP, ICRP and MPPD-Y&S.

The difference between DF_meas_ and DF_mod_ could potentially be explained by asymmetry in the breathing pattern, not reflected by the models. For the ICRP and NCRP models both sinusoidal and square waved breathing patterns were tested, showing minor differences in DF_mod_. Another factor, investigated in the earlier study from 2017, is the effect of inserting a pause in between breaths, but that could not explain the deviation between model and experiments. Furthermore, all breathing patterns were logged during the measurements and no consistent pauses in between breaths were observed.

**Fig. 3:**
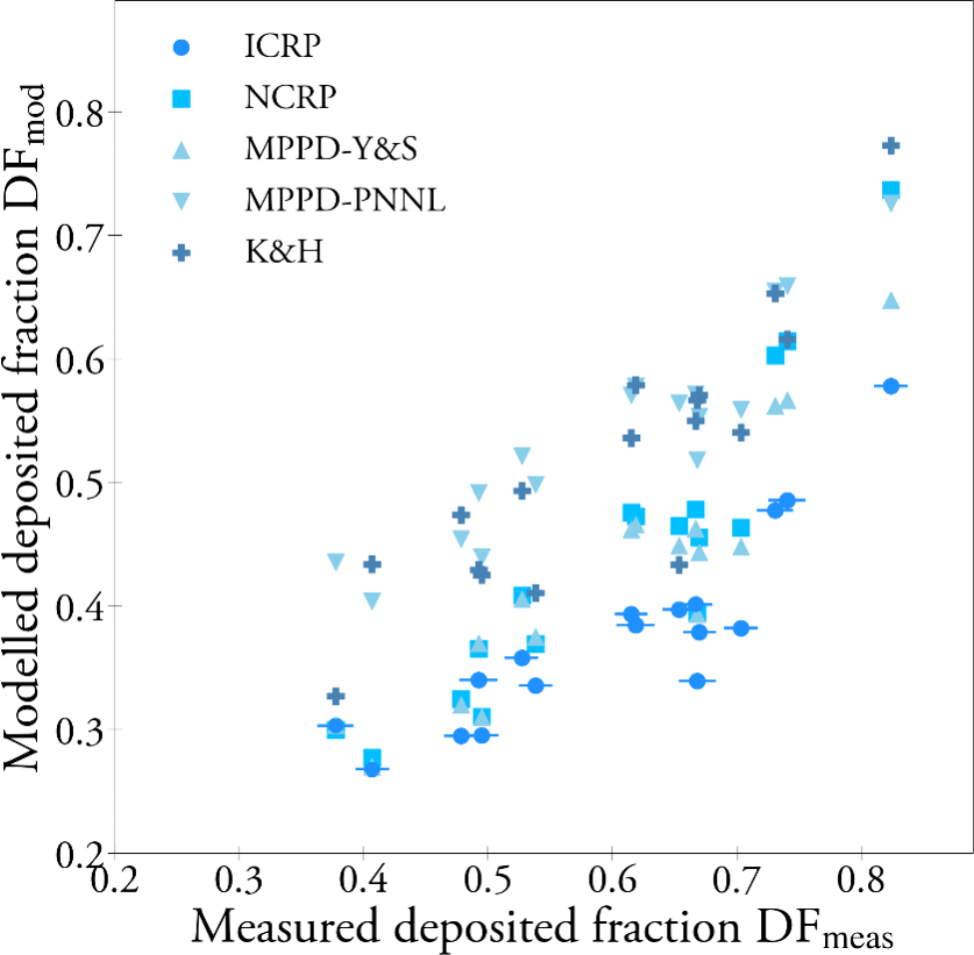
The modelled vs. measured DF. Error attributable to DF_meas_ are shown in the comparison to ICRP. Correlation plots for each model in separate panels and regressions lines are found in Additional file 2 (Figure A1)

The linear correlation between DF_meas_ and DF_mod_ for the individuals was significant for all models tested (p < 0.001), also when keeping FRC fixed (see Table 3; Fig. 3). In Fig. 3 also error bars representing the estimated experimental errors are shown. The largest errors were introduced by gradients in particle concentration and uncertainty of the particle losses in the set-up. In Figure A1, Additional file 2, correlation plots for each model in separate panels are shown, including a regression line. The strongest correlation between DF_meas_ and DF_mod_ was observed for MPPD using either of the two available lung models, with a slightly higher correlation for MPPD-PNNL (r = 0.93) compared to MPPD-Y&S (r = 0.92). If keeping FRC fixed, the correlation decreased slightly, see Table 3 (e.g. r = 0.89 for MPPD-PNNL).

**Table 3:** The average modelled DF_mod_ (± SD).

Model	DF_mod_ (± SD)	Alveolar deposition (% of DF)	Pearson’s *r*	P
ICRP	0.38 (± 0.08)	64 (± 6)	0.88	< 0.0001
NCRP	0.44 (± 0.12)	75 (± 6)	0.91	< 0.0001
NCRP_F_	0.43 (± 0.11)	75 (± 6)	0.89	< 0.0001
MPPD-Y&S	0.43 (± 0.10)	74 (± 6)	0.92	< 0.0001
MPPD-PNNL	0.54 (± 0.08)	65 (± 5)	0.93	< 0.0001
MPPD-PNNL_F_	0.53 (± 0.08)	65 (± 5)	0.89	< 0.0001
K&H	0.52 (± 0.10)		0.88	< 0.0001

Also shown is percentage of total DF_mod_ deposited in the alveolar region and Pearson’s correlation coefficient (*r*) describing the correlation between DF_mod_ with DF_meas_, with associated p-values indicated

### Correlation analysis of the difference between modelled and measured DF

**Fig. 4:**
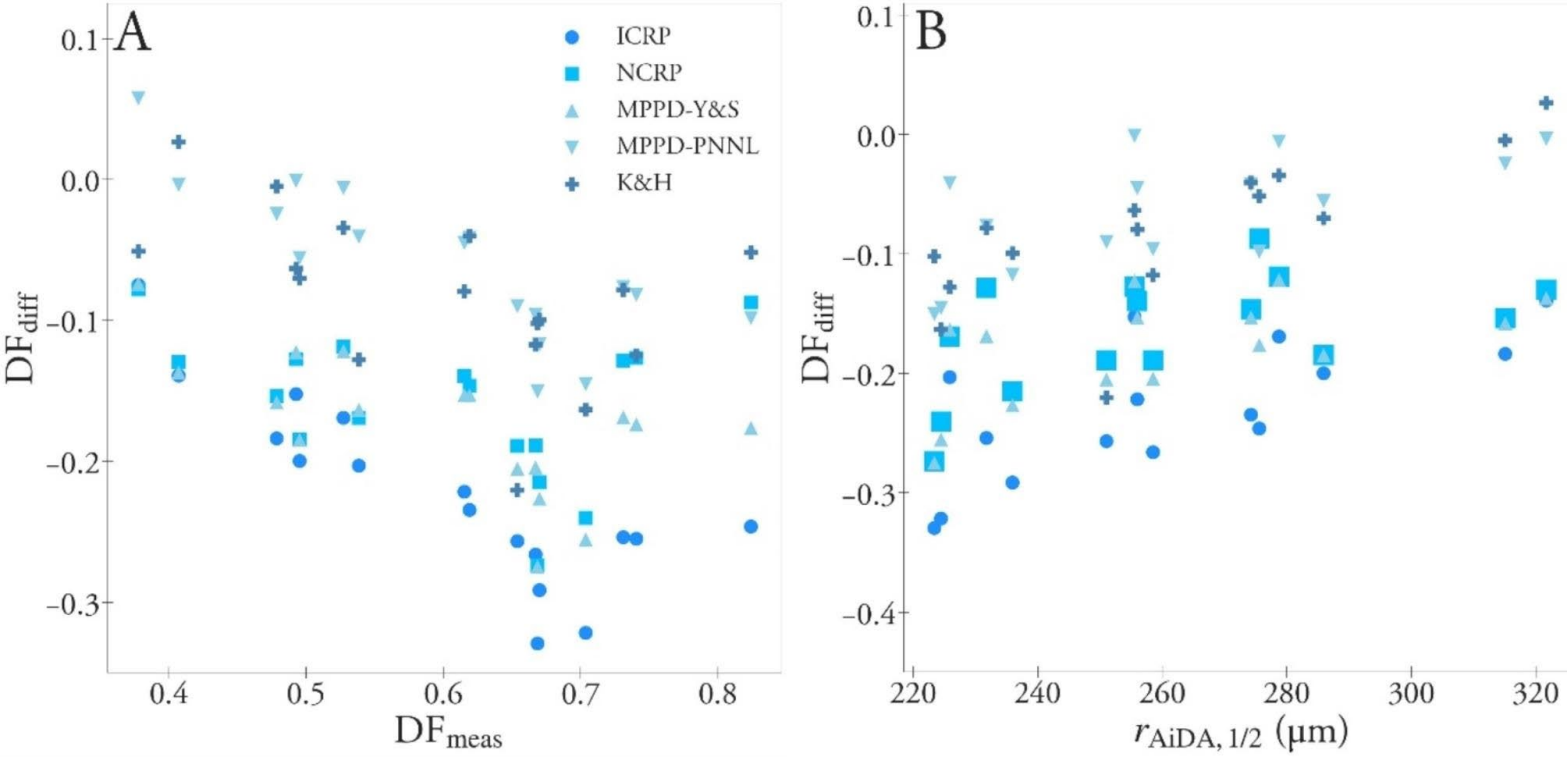
The difference between modelled and measured DF, DF_diff_, as a function of measured DF (DF_meas_) (left) and DF_diff_ as a function of *r*_AiDA,1/2_ (right)

The MPPD-PNNL model was the model that best predicted DF_meas_. For the subjects with the lowest DF_meas_, the model could well predict DF, but still underestimated DF for subjects with the highest DF_meas_ with ~ 20%, see Fig. 3 (and Figure A1d, Additional file 2). This is illustrated also in Fig. 4a where the difference between measured and modelled DF (DF_diff_ = DF_mod_ -DF_meas_) is shown as a function of DF_meas_.

To further investigate how the inter-subject variation in DF_meas_ was captured by the models, a Pearson correlation analysis was performed for DF_diff_ and lung characteristics. For models in Group A (individual FRC) DF_diff_ was significantly correlated (p < 0.01) with VC, FEV_1_, *r*_AiDA,1/2_, *V*_T_, and *T*_bc_. For Group B (fixed FRC), significant correlations (p < 0.01) with DF_diff_ was found for RV, FRC, oscillometry indices R_5_, and R_20_, and AiDA indices *r*_AiDA_, *r*_AiDA,1/2_ and R_0_ (Table A3 in Additional file 2). For all models, the strongest correlation with DF_diff_ was found for *r*_AiDA,1/2_, with the exception of MPPD-PNNL for which a slightly stronger correlation was found for VC. For models in Group A, multiple-linear regression analyses were performed for DF_diff_ including the variables that were significantly correlated with DF_diff_ (p < 0.01). Since FEV_1_ and VC are correlated, only VC was used as input to the regression models. Similarly, only *r*_AiDA,1/2_ was used as input to the model, while *r*_AiDA_ was not. VC and *r*_AiDA,1/2_ were selected since the correlation with DF_diff_ was stronger than for FEV_1_ and *r*_AiDA_. In the regression analysis the only significant correlation with DF_diff_ was found for *r*_AiDA,1/2_, which was significant for all models (Table A4 in Additional file 2). Replacing *r*_AiDA,1/2_ with *r*_AiDA_ in the regression model did not result in any significant correlation. Thus, again *r*_AiDA,1/2_ could best explain the difference between modelled and measured DF.

## Discussion

### Deposition measurements and comparison to models and lung variables

One of the purposes of the current study is to prove, or disprove, our earlier finding of significantly higher measured DF compared to those modelled using whole lung models for particles > 700 nm [1]. Such differences are important to investigate since, according to the experimental data, they would result in underestimations of the modelled deposited particle doses.

In the earlier study, the DF was measured using a set-up with a polydisperse aerosol. Using a polydisperse aerosol is an efficient way to measure DF over a range of particle sizes in parallel. The main drawback of such set-up is that errors are introduced by any slight shift in particle size between the inhaled and exhaled aerosol tanks (such as in the lungs). Another important improvement of the here used system is that the concentrations in the inhalation and exhalation tank are measured in parallel using two APSs. Thereby, the impact of concentration gradients over time is minimised. The difference in counting efficiencies of the two APSs are compensated for, and furthermore, the flowlines of the two APSs are switched during each measurement so that both APSs sample from the inhalation and exhalation tank, respectively.

The here reported average DF_meas_ (0.60 ± 0.12) is close, but somewhat lower than that reported for 2.3 μm particles in the earlier study for adults (0.63 ± 0.11) [1]. Apart from using different set-ups, the groups of participants were different in the two studies. Thus, the relatively small difference in average DF of the studies may be due to difference in lung function and anatomy of the subjects, respiratory parameters, or due to systematic experimental errors. A larger *V*_*T*_ is expected to result in higher DFs [2]. This is opposite to what is observed comparing the DFs of the earlier and present studies in relation to the average *V*_*T*_ in respective study (*V*_*T*_ = 0.94 L vs. 0.75 L in the current and previous study, respectively). The average FRC was higher in the current study than in the study from 2017 (3.9 L compared to 3.4 L). A larger FRC is known to result in lower DFs, which is in line with the observation that the DF reported in the current study is lower than in the previous. Most importantly, the trend of a significantly higher measured DF compared to those modelled is consistent in both studies.

Some of the experimentally reported DF from the 1980s [9, 20] was lower than those presented here. However, those DF measurements were performed for a small group of male subjects with typically larger lungs than the subjects in this study, likely explaining parts of the deviation. The somewhat later study by Bennett and co-authors [30], also reported relatively low DF for 2 μm particles compared to the current study (in average ~ 0.30). Later experimental studies report higher DF for coarse particles than those reported by both Heyder and co-authors and Bennett and co-authors, as for example the study by Kim co-authors [17] presenting just somewhat lower DF than here reported. In an older study by Giacomelli-Maltoni and co-authors [14] similar average DF was reported as in this and in our previous study [1, 2]. Even though part of the differences between the various experimental studies may be explained by different study groups (different lung function/lung anatomy and breathing patterns [31, 32], the large variation in the results points towards a considerable contribution from experimental limitations. There is no standard methodology for measurement of the respiratory tract deposition fraction (DF) of inhaled particles and many studies have overlooked critical methodological aspects that may have biased data [6]. In the current study, the system was optimized to minimize any foreseen experimental sources of errors.

Three whole lung models: ICRP, NCRP, MPPD and Y&S, were used to model the DF for each individual, using respiratory parameters (*T*_*bc*_, and *V*_*T*_) and FRC measured for each subject as input variables. The MPPD model, implemented using the recently developed PNNL lung model, was the model that best described the measured DF, both with respect to predicted average DF for the group, and had the highest correlation coefficients between predicted and measured DF for individual subjects. For the individuals with the lowest DF (~ 0.4), the predicted DF was very close to that measured, but the model underestimates the DF for subjects with higher DF_meas_ based on the breathing pattern and FRC.

Even though the MPPD-PNNL was the model best describing the measured DF for coarse particles, we have earlier shown that MPPD-Y&S well described the measured DF for submicron particles in the size range from 10 to 500 nm [1]. Since the DF predicted using the MPPD-PNNL model is higher in the submicron range than that predicted using the MPPD-Y&S (see Figure A1, Additional file 4), one could expect the MPPD-PNNL model to overpredict DF compared to those experimentally determined in the previous study of Rissler and co-authors. Note that the main deposition mechanism for particle in the diameter size range 10–400 nm is by diffusion, while for 2 μm particles by sedimentation [33].

Another observation made is that the individual variation predicted by all models was less than the individual variation found in the measurements. It is well established that the variation in DF to a large extent is driven by *V*_*T*_ and *T*_*bc*_ [17, 34], also confirmed by our study (see Table 2). However, the lung properties will also result in individual variation, but this have been scarcely studied. In earlier studies by Heyder and co-authors [20, 35] it was suggested that the volume of the breath that reaches the peripheral lung, and the residence time of the air in the peripheral lung, are two important parameters explaining the variation in DF. In our earlier study from 2017 we show that, apart from breathing pattern, anatomic dead space (and R_5_ output from oscillometry) could better describe the individual variation than FRC [2]. The observed correlation of DF with anatomic dead space is in line with the suggestion of Heyder and co-authors since the fraction of the *V*_*T*_ reaching the peripheral lung is proportional to anatomic dead space.

In both the semi-empirical regional compartment models (e.g. ICRP and NCRP) and the multiple path model (MPPD), FRC is the lung property that is used to scale lung size. From the correlation analysis we see that including the FRC of each subject does improve the correlation between modelled and measured DF on an individual basis. However, there is still variations in DF that are not explained by neither breathing pattern nor FRC.

As stated already in the study by Heyder et al. [20], and confirmed by results from the models applied in the current study (see Table 3), 2 μm sized particles are mainly deposited in the peripheral lung by sedimentation (at relaxed breathing at rest). The rationale behind including the new AiDA technique as a lung function test in the current study is that *r*_AiDA_ is a lung function variable that could provide a measure of airspaces in the acinar region of the lungs, and thus should correlate with the lung deposition of the 2 μm particles (mainly deposited in the peripheral lung). From the correlation analysis we see that *r*_AiDA,1/2_ correlate with DF_meas_, and in fact has the strongest correlation, also when including *V*_*T*_ and *T*_*bc*_ as variables in the multiple regression analysis. This fact indicates that *r*_AiDA,1/2_ is indeed measuring the distances in the peripheral lung and that *r*_AiDA,1/2_ could provide crucial information needed to explain the individual variation in the deposited fraction of the inhaled particles.

To further investigate this, we looked at the correlation between lung function variables and the difference in modelled and measured DF (DF_diff_, here defined as DF_mod_ - DF_meas_). This step was performed to see if any measured lung function indices could explain the remaining variability after accounting for breathing variables in the models, and for some models also FRC. Even though *V*_*T*_ and *T*_*bc*_ were used as input to the models, DF_diff_ correlated negatively with *T*_*bc*_ and *V*_*T*_ for ICRP and MPPD-PNNL (Table A3, Additional file 2). This could indicate that the models overestimate the effect of *T*_*bc*_ and *V*_*T*_. The strongest correlation with DF_diff_ was found to be that with *r*_AiDA,1/2_, which is a measure of the radius of airspaces in primarily the acini at normal breathing. As expected, FRC (and thus VC) correlated with DF for results modelled without using an individually varying FRC as model input.

Multivariate linear regression models were applied to explain DF_diff_ for the models where FRC was varied for each individual (group A). In the analysis we only included the variables that were significantly correlated to DF_diff_ for any of the models in each group (p < 0.01), which for group A were VC, FEV_1_, *r*_AiDA,1/2_, *T*_bc_, and *V*_T_. As can be seen from the results (given in Table A4 in Additional file 2), *r*_AiDA,1/2_ can significantly explain the difference between the measured and modelled DF, further supporting that *r*_AiDA,1/2_ is a measure of the lung properties that explains much of the individual variation.

The lung models used in whole lung deposition models are often based on computer tomography (CT) of one or very few lungs. These are then typically scaled by FRC to account for individual variation of lung size. It is not possible to numerically model the distal airway generations based on CT images [11]. Lung diseases and age is known to cause a change in distal airspace size [36], an effect that has been shown to be measurable with AiDA [37, 38]. We here show that accounting for variation in the structure/dimensions of the peripheral lung, here assessed with AiDA (*r*_AiDA,1/2_), in particle deposition modelling could presumably improve models to capture difference in DF – on a group level (e.g. age groups or groups with specific lung diseases) and for individuals. Such improvement could be important, especially in the area of applying whole lung models to estimate lung deposition of inhaled drugs.

### AiDA at half inflation

In this study, the *r*_AiDA_ was for the first time measured at half inflation (½ VC), motivated by that this would provide peripheral airspace dimensions closer to those at normal relaxed breathing. Breathing pattern logged before and during the inhalation manoeuvre in the AiDA instrument confirms that the lung inflation at ½ VC is very close to FRC+½ *T*_v_. This could be one reason why *r*_*AiDA,1/2*_ (at half inflation) has a stronger correlation with DF than r_AiDA_ at full inflation, explained by for example non-uniform ventilation of the lung depending on the inflation volume.

As expected, the measured *r*_AiDA,1/2_ was typically lower than *r*_AiDA_ (280 ± 36 μm compared to 261 ± 36 μm), with a ratio of 1.11. Assuming a symmetric expansion of the lung, the expected ratio was estimated to 1.09, based on RV and the volumes inhaled during the AiDA inhalation manoeuvre (see Eq. 3). Despite the small difference expected in AiDA airspace dimensions at full and half inflation, the measured *r*_AiDA_ values agree with the expected difference on a group level. This observation strengthens the hypothesis that *r*_AiDA_ is indeed a measure of the average radius of the airspaces in the acinar region of the lungs [38].

It is not yet fully concluded what *R*_0_ is a measure of, but one hypothesis is that it reflects geometric properties, such as heterogeneity, of small conducting airways. The average *R*_*0*_ measured at half inflation was significantly lower than that measured at full inflation, which agrees with earlier observations [39]. The observed decrease in *R*_0_ can be explained by less deposition in the conducting airways at a shorter and shallower breathing cycle. However, *R*_0_ measured at full inflation has earlier been shown also to correlate with measures of lung heterogeneity [38, 40]. The decreased *R*_*0*_ at the smaller inhaled volume with particles could also be due to a larger influence of entrainment and mixing with particle free air from the residual volume, which would strengthen the hypothesis that *R*_*0*_ is a measure of lung heterogeneity.

### Study limitations

In this study we had considerably fewer subjects than in the earlier study [1, 2], motivated by the fact that the main purpose of the study was not to explain the individual variation in lung deposited fraction. Still, we can show that the DF was significantly correlated with several lung function variables. We attempted to do a Principal component analysis, PCA (as in the earlier study), but the number of subjects was too small. We also intended to include anatomic dead space as a lung function variable but due to a malfunctioning instrument this variable was left out.

### Estimation and discussion of experimental errors

The set-up was built according to the criteria given and discussed by Löndahl and co-authors [6]. The experimental set-up to measure the DF was carefully designed to minimize any foreseen systematic or randomized errors (optimizing precision and accuracy in the measurement). This is the reason for using a monodisperse aerosol. Furthermore, the system was designed to minimise particle losses and the flow lines from the inhalation and exhalation tank were made symmetric in all aspects (e.g. dimensions, length, number of bends, valves, driers) to have as similar particle losses as possible. The dead space volume of the mouthpiece was minimized and further corrected for (minimized to reduce the correction and potential errors) as described by Eqs. 1 and 2. In the earlier set-up only one APS was used. The major reason for this was to avoid any error due to instrumental differences. However, when having only one instrument, alternating between sampling from the inhalation and exhalation tank, any drift in particle concentration over time can introduce a substantial error in the determined DF. Therefore, two APSs were used in the current experimental set-up.

The estimated errors are shown as error bars in Fig. 3. The error is estimated considering several factors, added assuming that they are uncorrelated. The factors included in the error propagation are gradients in the particle concentration, variations in APS counting efficiency, uncertainty in the loss calibration curve, non-isokinetic sampling efficiency, and factors related to uncertainties in breathing variables, including breathing frequency determined by the algorithm, tidal volume measured by the pneumotachograph, and effects of the periodization. The error in tidal volume and breathing frequency was translated to an error in DF according to their respective effect on DF as predicted by the model described in the study by Kim and Hu [17]. The largest errors were introduced by uncertainty in the determination of instrumental particle losses and gradients in particle concentration, due to the time it takes for the aerosol to pass from the inhalation sampling point to the point of the sampling from the exhalation tank (residence time in the lungs and in the system was not considered in the data analysis).

## Conclusions

In this study, we have designed an experimental set-up to measure the particle deposition in the respiratory tract, carefully considering the design to minimize the potential errors, and measured the deposition of 2.3 μm particles (aerodynamic diameter) in seventeen healthy adults (22–68 years). The particle size used represent coarse particles in a size range that predominantly is deposited in the peripheral lung by sedimentation, during tidal breathing at rest. The average measured DF was 0.60 ± 0.14, which is significantly higher than the DFs predicted by any of the models tested (NCRP, ICRP and MPPD), which range from 0.37 ± 0.08 to 0.53 ± 0.09. All models predicted a smaller variation in DF between individuals than measured. The MPPD model, implemented using the recently developed PNNL lung model, was the model that best described the measured DF, both with respect to average DF of the group and at an individual level as it provided the best correlation between predicted and measured DF for individual subjects. Still, the model underestimated the DF for the subjects with the highest DF_meas_. The deviations between model and measurements show that there is a need of improvement for whole lung models.

Pearson’s correlation analysis and multivariate linear regression analysis show that, apart from VT and Tbc, the main individual variability in measured particle deposition was best explained by breathing pattern and *r*_AiDA,1/2_. Our results support the hypothesis that *r*_AiDA,1/2_ is a measure of distal lung airspace size at tidal breathing and that measurements of the distal lung airspaces (using the modified AiDA method) can be used to better understand the individual variation in the dose of particles to healthy and diseased lungs.

## Method section

### Study design

The study included 17 healthy adults, comprising 9 men and 8 women aged 21–68 years. The respiratory tract deposition was measured during normal and spontaneous breathing through a mouthpiece, while seated in a relaxed position. Each participant performed a lung function test on a separate occasion. Two participants did not complete all lung function tests, leaving 15 participants (9 men, 6 women) with a full data set for the correlation analysis. The study was approved by the Swedish Ethical Review Authority, dnr 2019–04770, and was performed in accordance with the Declaration of Helsinki. All participants gave their written informed consent.

### Experimental set-up

The set-up for measurement of respiratory tract deposition of inhaled particles was based on the same principle as in earlier systems [e.g. 1, 2, 41, 33], but with modifications made to minimise potential errors. The principle of the set-up follows the general experimental guidelines described in Löndahl et al. [6].

**Fig. 5:**
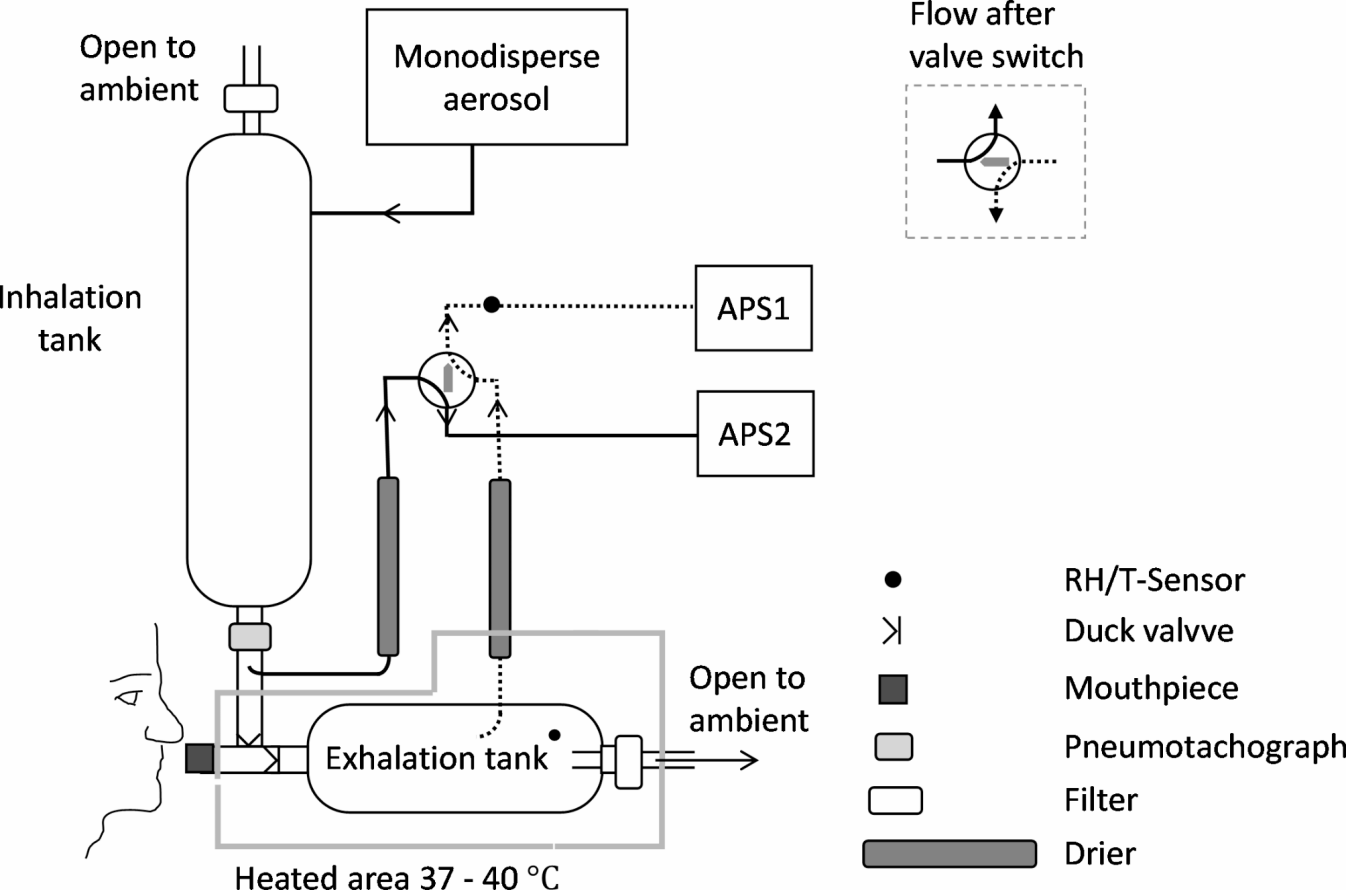
Schematic drawing of the experimental set-up for lung deposited particle fraction. Arrows indicate the flows through the system. The solid line represents the sampling line for inhaled aerosol whereas the dashed line represents the sampling line for exhaled aerosol

The set-up consisted of three parts: an aerosol generation module, an inhalation system and particle detection. An overview of the system is given in Fig. 5.

#### Aerosol genseration

Measurements were performed for monodisperse particles of ~ 2 μm. The particles consisted of spherical, monodisperse, and amorphous^1^ SiO_2_ with a geometrical diameter of 1.7 μm (Micro Particle GmbH SiO2-R-2.0, 5% v/w). The particles were generated from a solution of 1.3% v/w SiO_2_ in MQ-water using an atomizer (Condensation Aerosol Generator SLG270, Topas GmbH, Germany). The droplets were dried with a diffusion drier, and further mixed with particle free dry air (~ 6 L/min) to achieve a high enough flow of ~ 10 L/min with an RH < 20%. The particles passed a neutraliser before injection into the inhalation tank. The geometrical size of 1.7 μm correspond to an equivalent aerodynamic diameter (*d*_*ae*_) of 2.3 μm when using the density of the particles according to the manufacturer of 1.85 g/cm^3^. This was confirmed by measurement with an aerodynamic particle sizer (APS, model 3321, TSI Inc.) showing a *d*_*ae*_ of 2.3 μm and geometrical standard deviation of 1.04. Double and triple spheres were minimised by diluting the solution, and at the dilution rate used no double or triple spheres could be detected. The generated size distribution is shown in Figure A1 in Additional file 3. The particle generation was stable with a relative standard deviation for each 5-minute period of typically 2–5%, and with particle concentrations of ~ 50 particles/cm^3^.

#### Inhalation system

The inhalation system consisted of two tanks (one for inhaled air and one for exhaled air), duck valves, a pneumotachograph for logging of inhaled and exhaled flows, temperature (T) and relative humidity (RH) sensors, and a mouthpiece (see Fig. 5). The generated aerosol was continuously injected into the inhalation tank with a flow rate of ~ 10 L/min. Both tanks were made of stainless steel to avoid deposition by electrical charge and had sufficiently large volumes and geometries to obtain mixing of the breaths: 10 L for the tank with inhaled air and 5 L for exhaled air.

The breathing flow through the system was directed by the duck valves. During exhalation, the excess aerosol from the generation was wasted to a filter connected to room air. The mouthpiece was designed to minimise instrumental dead space (volume between the duck valves, including the mouthpiece). The inhalation flow was monitored with a time resolution of 0.1 s using a pneumotachograph and logged by a customized software (written in LabVIEW 2018, NI Inc.).

#### Particle detection

The inhaled and exhaled particle concentrations were measured in parallel by continuous sampling by two aerodynamic particle sizers (APS, model 3321, TSI Inc.) at a sampling flow rate of ~ 1 L/min. The sampling point were chosen as close to the mouthpiece as possible from the inhaled aerosol flow, while from the exhalation flow the sampling was made further down in the tank in order to allow the exhaled air to mix before sampling (air from the deep lung mixed with that from the upper airways). The exhalation tank was heated to 37–40 degrees to avoid condensation of water and the aerosol was dried before entering the APS. The sampling line from the inhalation and exhalation tanks were made as identical as possible (same tube length, diameter, number of bends, types of valves and tubing) and were equipped with identical driers to get similar particle losses and pressure drops for both samples. RH and temperature were monitored continuously in the sampling lines. The particle size distributions were the same before and after passing through the sampling line and dryer.

APS counting efficiency is known to vary slightly between instruments and the APS instrument requires regular calibration [42]. To control and minimize bias from any differences in APS counting efficiency between the two APSs, a four-way valve was used to switch between the instruments so that both instruments sampled from both inhalation and exhalation tanks at some point during the measurement as further described in Sect. 5.4. Furthermore, the two APS instruments were running in parallel, sampling the same aerosol, before and after each measurement, to assure stability and well-functioning of the instruments. A difference in the counting efficiency between the two APS instruments was observed (20%), mainly explained by difference in the sampling flows. However, the off-set was stable over time and could be accounted for.

### Characterizing the set-up

The pneumotachograph was calibrated before, after and once during the period that the measurements took place. The calibration showed that the same polynomial fit (of degree two) could be used during the whole period to translate the obtained raw voltage signal of the pressure sensor to the corresponding flow being measured.

The particle losses, from the point of aerosol sampling from the inhalation to the point of sampling from exhalation tank, was characterised and compensated for. The loss characterisation was performed at constant flows ranging from 5 to 14 L/min and repeated at three different occasions (before, during and after the study). The results are shown in Additional file 3 (Figure A2). The use of constant flow during the loss calibration was motivated by tests and calculations showing that the main mechanism of particle losses in the system was by sedimentation, and that sedimentation primarily depends on residence time (linearly proportional to the flow). Losses by impaction was negligible in the relevant flow ranges due to the large dimensions of the flow paths (equivalent to the USP throat and thoroughly characterized earlier [43, 44]. This was further confirmed by the results from the characterization of the particle losses that were decreasing linearly with the flow rate.

At ventilation rates from 5 to 14 L/min, the system intrinsic losses varied from 4 to 10%, with higher losses at lower flow rates. At the average *V*_*e*_ of the subjects (8.8 L/min), the losses were 7.3%. Note that the losses compensated for include losses in the inhalation system between the sampling point of the inhaled flow (just after the pneumotachograph) and the sampling point from the exhalation tank. There are additional losses also in the symmetric sampling lines between the tank and the APS, determined to ~ 10–15%, but since the system was built with symmetric sampling lines, these losses did not affect the relative difference between inhaled and exhaled aerosol concentration, and thus measured DF.

The dead space of the mouthpiece (31.4 mL) was corrected for according to Eq. 2. If not adjusted for, the dead space of the mouthpiece would have resulted in an underestimation of the DF by ~ 3–5%, depending on *V*_*T*_.

### Measurement procedure and data analysis

The measurements were carried out with the subject sitting in a relaxed position during tidal breathing through a mouthpiece. A nose clip was used to assure breathing through the mouth. Each measurement took in total 16 min, where the first minute was wasted to allow the system to equilibrate. Both APS instruments sampled in parallel during the whole period, with one APS sampling from the inhaled aerosol flow and one from the exhalation tank. The 4-way valve was switched every 5th minute so that the APS that first sampled from the inhaled flow later sampled from the exhaled flow, and vice versa. The switching events resulted in 3 periods, each period being 5 min long. The procedure was developed to minimise errors due to gradients in the concentrations and due to any change in APS counting efficiency (for example due to clogging of the APS nozzle, or water condensation).

To calculate the true respiratory tract deposition fraction from the measurements, we accounted for (1) the particle losses in the system and (2) the dead space in the mouthpiece according to (modified from Löndahl et al. [3]:

1 $${DF}_{\text{m}\text{e}\text{a}\text{s}}={CF}_{\text{m}\text{o}\text{u}\text{t}\text{h}\text{p}\text{i}\text{e}\text{c}\text{e}}\left(1-\frac{{C}_{ex}}{{C}_{in}\bullet (1-{DF}_{\text{e}\text{q}\text{u}\text{i}\text{p}}\left(Q\right))}\right)$$

where *C*_in_ and *C*_ex_ are the aerosol concentrations in the inhalation tank and exhalation tank, respectively, and *DF*_equip_ is the particle losses due to deposition in the system at the specific gas flow (Q) and *CF*_mouthpiece_ is the correction factor to account for the dead space in the mouthpiece given by.

2 $${CF}_{\text{m}\text{o}\text{u}\text{t}\text{h}\text{p}\text{i}\text{e}\text{c}\text{e}}=\frac{{V}_{T}}{{V}_{T}-{V}_{mouthpiece}}$$

### Lung function tests

Each participant performed a comprehensive lung function test including forced oscillatory technique (FOT), spirometry and the recently developed AiDA-method. The parameters measured were, vital capacity (VC), forced expiratory volume in 1 s (FEV_1_), total lung capacity (TLC), residual volume (RV), functional residual capacity (FRC), and diffusing capacity for CO (D_L,CO_), all measured according to current guidelines [45–47] using Masterscreen Body, Viasys GmbH - Erich Jaeger, Hoechberg, Germany. The lung function data are presented in Table 1. Corresponding tables with data for every individual are found in Additional file 1 (Table A1).

Respiratory system resistances at 5 Hz (R5) and at 20 Hz (R20) were measured with FOT. The respiratory system resistance is interpreted to be related to central and peripheral airway calibre (5 Hz for central airways and 20 Hz for peripheral airways). FOT was performed according to the recommendations by Oostveen et al. [48] with three consecutive measurements. The subject supported the cheeks during the measurement and was instructed to breathe normally. A nose clip was applied to prevent breathing through the nose.

In addition to the respiratory resistances, reactance at 5 Hz (X5), area of reactance (AX) and resonant frequency (fres) were also derived from the FOT measurements. These variables have been interpreted to be related to respiratory stiffness [48, 49], respiratory system elastic properties [50], and chest size and tissue composition [50], respectively.

### Airspace dimensions assessment with the AiDA method

Airspace dimension assessments (AiDA) measurements were made with a set-up that has been described in detail elsewhere [21]. With the AiDA method, peripheral airspace dimensions are measured as diffusional distances in the distal lung. The airspace dimensions are derived from measurement of deposition of aerosol nanoparticles in the lungs during a specified procedure of a few consecutive measurements with varying breath-hold times [22]. A measurement provides an average radius of the airspaces in primarily the alveolated region of the lungs *r*_AiDA_ [38], and an imaginary zero-seconds recovery, *R*_0_, which is assumed to provide information on small conducting airways [22].

AiDA measurements are normally performed with full inflation of the lung. However, in this study we also perform the AiDA measurement procedure after inhaling half vital capacity, as this presumably would provide peripheral airspace dimensions closer to those during normal breathing. Assuming a symmetric expansion of the lung (i.e. that the expansion of all airways are made according the same percental increase in volume compared to initial volume), the theoretical difference in *r*_AiDA_ at full and half VC can be estimated by:

3 $$\frac{{r}_{\text{A}\text{i}\text{D}\text{A}}}{{r}_{\text{A}\text{i}\text{D}\text{A},1/2}}=\sqrt[3]{\frac{RV+{VC}_{\text{A}\text{i}\text{D}\text{A}}}{RV+{\frac{1}{2}VC}_{\text{A}\text{i}\text{D}\text{A}}}}$$

where *VC*_AiDA_ and ½ *VC*_AiDA_ corresponds to the inhaled volume during the AiDA measurements at full inflation and half VC, respectively, and RV is the residual volume.

### Modelling DF

Particle deposition in the respiratory tract was modelled using available whole lung models: the Multiple-Path Particle Dosimetry model (MPPD©, Applied Research Associates, Inc., Albuquerque, NM, USA) [26, 27], ICRP [24] and NCRP [25]. Also, a simplified empirical model was tested [17]. The models include particle deposition in the extrathoracic region, in this case oral deposition. The deposition in the extrathoracic region was modelled as prescribed in the publications for the respective model. Apart from that the models use different algorithms and different lung geometries, there are also differences in how they are practically implemented.

The ICRP and NCRP are single path regional compartment models, here implemented through the software Mimetikos Preludium™ v1.2.0.0 [51]. For the NCRP deposition model we used the symmetric lung geometry from Yeh and Schum [28], while empirical functions from ICRP were used to calculate deposition for the ICRP model [24]. In the Mimetikos Preludium software the initial size of the lung is scaled according to the FRC entered in the model. However, no geometrical data in the alveolar ducts and sacks are used, the size of these are kept constant regardless of the FRC entered in the model while the number of alveoli is increased with FRC. A breath is simulated by expansion and contraction of the airway diameters. A sinusoidal inhalation curve was used to simulate varying inspiratory and expiratory flow rates over time (peak expiratory and inspiratory flow rates were set equal). Deposition was calculated using bolus probes with 30 segments covering the bolus volume, which was set equal to *V*_*T*_.

The MPPD model incorporates lung anatomy models with asymmetric representation of the major segmental bronchi leading to the five lung lobes (each represented by a symmetric model) and calculates the deposition for each airway using a constant lung volume of FRC + *V*_T_/2 and a square waved breathing pattern (constant inhalation and exhalation flow). The deposition was modelled with MPPD v3.04 with two different lung anatomy models available through the software: the asymmetric PNNL (Pacific Northwest National Laboratory) [29] model and the Yeh and Schum 5-lobe model [28].

The regional deposition was modelled for each subject using *V*_T_ and *T*_bc_ from the deposition measurements, and the individual FRC from the lung function tests. The upper respiratory tract (URT) volume for each subject was scaled by FRC according to ICRP and used in all deposition models. The deposition was modelled for oral breathing without inhalability adjustment, for upright position, for an entire breath (inspiration + expiration) without pause. Additionally, DF was modelled using a fixed FRC (set to the average measured FRC, 3865 mL) using NCRP and MPPD-PNNL. The main purpose was to disentangle the effect of breathing pattern from sizing of the lung. For the same reason, total DF was modelled using a simplified empirical model, accounting for variations in DF only due to breathing pattern (*V*_T_ and *T*_bc_), not any sizing of the lung nor lung intrinsic properties [17].

### Statistical analysis

Means with associated standard deviations (± SD) were used to describe reported values. Pearson’s linear correlation test was used to determine associations between variables. A probability value (p-value) of 0.05 was set to indicate the level of statistical significance.

Analysis of variance (ANOVA) showed that the average DF, measured and modelled, were different (p < 0.0001). Paired samples t-test was used to compare differences between repeated values for the same subjects and to compare DF_meas_ with DF_mod_ for each model used.

Correlation analysis was used to determine which variables that influenced DF_meas_ and the difference between DF_mod_ and DF_meas_: DF_diff_ for each model. The correlation analysis was followed-up by linear multivariate analysis to investigate which variables that could be used to predict the DF_diff_.

### Electronic supplementary material

Below is the link to the electronic supplementary material.

**Additional file 1.** Table with data for all individuals recruited, showing background variables, breathing parameters, lung function variables. **Additional file 2.** Complementing tables and figures with results from the statistical analysis. **Additional file 3.** Complementing data from the deposition measurements showing particle properties and internal instrumental losses. **Additional file 4.** Modelling the DF for data from Rissler et al., 2017b.

## Data Availability

All data generated or analysed during this study are included in this published article and its supplementary information files.

## Footnotes

1 The crystalline fraction was determined using X-ray diffraction (XRD) to below 1%.

## Abbreviations

DF_diff_: Difference between DF_mod_ and DF_meas_ (DF_mod_ DF_meas_)
DF_meas_: Measured total deposited fraction
DF_mod_: Modelled total deposited fraction
FEV_1_: Forced expiratory volume in 1 s
FRC: Functional residual capacity
*R*_0_: Zero seconds recovery at TLC derived from AiDA
*R*_0,1/2_: Zero seconds recovery derived from AiDA after inhalation of half VC
R_20_: Respiratory resistance at 20 Hz from oscillometry
R_5_: Respiratory resistance at 5 Hz from oscillometry
*r*_AiDA_: Airspace size derived from AiDA measurements at TLC
*r*_AiDA,1/2_: Airspace size derived from AiDA measurements AiDA after inhalation of half VC AiDA
RV: Residual volume
*T*_bc_: Time of breath cycle
TLC: Total lung capacity
VC: Vital capacity
VC_AiDA_: Vital capacity during the AiDA measurements
*V*_e_: Minute volume ventilation rate
*V*_T_: Tidal volume
