# Supplementary material for: An experimental study on lung deposition of inhaled 2 μm particles in relation to lung characteristics and deposition models
Source: Part Fibre Toxicol. 2023 Oct 24;20:40. doi: 10.1186/s12989-023-00551-9 (PMC10594870; doi:10.1186/s12989-023-00551-9)
Supplement: Supplementary file 1 — Additional file 1. Table with data for all individuals recruited, showing background variables, breathing parameters, lung function variables. Additional file 2. Complementing tables and figures with results from the statistical analysis. Additional file 3. Complementing data from the deposition measurements showing particle properties and internal instrumental losses. Additional file 4. Modelling the DF for data from Rissler et al., 2017b. [file 12989_2023_551_MOESM1_ESM.docx]

Additional file 1

**Subject data**

**Table A1.** Data for all individuals recruited showing background variables, breathing parameters, lung function variables, % of predicted for selected variable (according to Quanjer et al., 1993), and DF_meas_.

| Subject number | 1 | 2 | 3 | 4 | 5 | 6 | 7 | 8 | 9 |
| --- | --- | --- | --- | --- | --- | --- | --- | --- | --- |
| Age (years) | 30 | 30 | 36 | 40 | 41 | 44 | 45 | 50 | 52 |
| Height (cm) | 175 | 175 | 183 | 183 | 182 | 173 | 200 | 183 | 172 |
| Weight (kg) | 60 | 64 | 89 | 76 | 94 | 72 | 118 | 70 | 67 |
| *V*_T_ (L) | 0.68 | 0.62 | 1.32 | 1.10 | 1.02 | 0.70 | 1.95 | 1.03 | 0.68 |
| *T*_bc_ (min) | 0.085 | 0.083 | 0.159 | 0.104 | 0.101 | 0.075 | 0.251 | 0.118 | 0.077 |
| *V*_e_ (L/min) | 8.0 | 7.5 | 8.3 | 10.6 | 10.1 | 9.4 | 7.8 | 8.8 | 8.9 |
| DF_meas_ | 0.65 | 0.54 | 0.73 | 0.67 | 0.67 | 0.49 | 0.82 | 0.67 | 0.49 |
| *r*_AiDA_ (µm) | 299 | 229 | 255 | 288 | 274 | 266 | 334 | 238 | 303 |
| *R*_0_ (a.u) | 0.42 | 0.50 | 0.48 | 0.61 | 0.71 | 0.64 | 0.48 | 0.74 | 0.85 |
| *r*_AiDA,1/2_ (µm) | 251 | 226 | 232 | 236 | 258 | 255 | 276 | 223 | 286 |
| R_0,1/2_ (a.u.) | 0.31 | 0.18 | 0.27 | 0.60 | 0.51 | 0.28 | 0.52 | 0.59 | 0.50 |
| R_5_ (KPa s/L) | 0.51 | 0.36 | 0.31 | 0.34 | 0.34 | 0.36 | 0.28 | 0.22 | 0.33 |
| R_20_ (KPa s/L) | 0.5 | 0.3 | 0.26 | 0.33 | 0.34 | 0.37 | 0.22 | 0.21 | 0.33 |
| DLCO | 7.1 | 7.7 | 12.3 | 10.6 | 10.8 | 7.2 | 12.4 | 10.6 | 7.3 |
| TLC (L) | 5.5 | 5.9 | 7.5 | 7.8 | 7.7 | 5.7 | 9.2 | 8.4 | 6.8 |
| FRC (L) | 2.3 | 3.2 | 3.4 | 4.1 | 3.3 | 3.3 | 3.3 | 5.6 | 4.3 |
| FEV_1_ (L) | 3.2 | 3.9 | 4.8 | 4.4 | 4.8 | 3.2 | 6.1 | 3.9 | 3.5 |
| VC (L) | 4.1 | 4.4 | 5.8 | 5.8 | 5.9 | 4.0 | 7.1 | 5.5 | 4.6 |
| RV (L) | 1.4 | 1.5 | 1.7 | 1.9 | 1.8 | 1.8 | 1.9 | 2.9 | 2.2 |
| FEV_1_/VC (%) | 78 | 87 | 83 | 76 | 81 | 81 | 86 | 70 | 76 |
| TLC (% pred.) | 96% | 103% | 99% | 103% | 103% | 101% | 104% | 112% | 122% |
| FRC (% pred.) | 78% | 108% | 96% | 116% | 94% | 111% | 83% | 154% | 146% |
| FEV_1_ (% pred.) | 89% | 107% | 110% | 105% | 114% | 101% | 126% | 97% | 119% |
| VC (% pred.) | 101% | 107% | 109% | 113% | 115% | 108% | 117% | 112% | 135% |
| RV (% pred.) | 85% | 93% | 90% | 93% | 87% | 96% | 82% | 130% | 115% |
| FEV_1_/VC (% pred.) | 93% | 105% | 103% | 95% | 101% | 100% | 109% | 89% | 95% |

Abbreviations: *V*_T_ tidal volume; *T*_bc_ time of breath cycle; *V*_e_ minute volume ventilation rate; DF_meas_ measured total deposited fraction; *R*_0_ zero seconds recovery and *r*_AiDA_ airspace size derived from AiDA measurements at TLC and *R*_0,1/2_ and *r*_AiDA,1/2_ are from AiDA measurements at half inflation. R_5_ and R_20_ respiratory resistance at 5 and 20 Hz from oscillometry, respectively; TLC total lung capacity; FRC functional residual capacity; FEV_1_ forced expiratory volume in 1 s; RV residual volume; VC vital capacity.

Cont. Table A1

| Subject number | 10 | 11 | 12 | 13 | 14 | 15 | 16 | 17 |
| --- | --- | --- | --- | --- | --- | --- | --- | --- |
| Age (years) | 53 | 61 | 62 | 62 | 65 | 68 | 21 | 56 |
| Height (cm) | 175 | 178 | 179 | 176 | 190 | 180 | 167 | 166 |
| Weight (kg) | 73 | 82 | 77 | 72 | 82 | 76 | 69 | 75 |
| *V*_T_ (L) | 0.96 | 0.87 | 0.71 | 0.96 | 1.05 | 0.78 | 0.48 | 1.15 |
| *T_bc_* (min) | 0.108 | 0.086 | 0.076 | 0.104 | 0.129 | 0.094 | 0.058 | 0.151 |
| *V*_e_ (L/min) | 8.9 | 10.0 | 9.4 | 9.2 | 8.1 | 8.3 | 8.3 | 7.6 |
| DF_meas_ | 0.70 | 0.53 | 0.41 | 0.62 | 0.62 | 0.48 | 0.38 | 0.74 |
| *r*_AiDA_ (µm) | 233 | 312 | 307 | 233 | 310 | 318 | NA | NA |
| *R*_0_ (a.u) | 0.42 | 0.58 | 0.85 | 0.33 | 0.58 | 0.73 | NA | NA |
| *r*_AiDA,1/2_ (µm) | 224 | 279 | 322 | 256 | 274 | 315 | NA | NA |
| R_0,1/2_ (a.u.) | 0.28 | 0.61 | 0.46 | 0.49 | 0.40 | 0.57 | NA | NA |
| R_5_ (KPa s/L) | 0.36 | 0.42 | 0.25 | 0.42 | 0.27 | 0.25 | 0.32 | 0.59 |
| R_20_ (KPa s/L) | 0.37 | 0.38 | 0.23 | 0.4 | 0.28 | 0.24 | 0.32 | 0.45 |
| DLCO | 7.5 | 7.4 | 9.0 | 7.0 | 11.3 | 10.5 | NA | NA |
| TLC (L) | 7.1 | 6.7 | 8.5 | 6.5 | 8.1 | 8.2 | NA | NA |
| FRC (L) | 3.8 | 3.6 | 5.1 | 3.4 | 4.4 | 5.1 | NA | NA |
| FEV_1_ (L) | 3.8 | 3.1 | 3.4 | 3.2 | 3.7 | 3.5 | 3.5 | 3.4 |
| VC (L) | 4.7 | 4.4 | 5.6 | 4.4 | 5.2 | 5.1 | 3.8 | 4.5 |
| RV (L) | 2.3 | 2.4 | 2.9 | 2.2 | 2.9 | 3.1 | NA | NA |
| FEV_1_/VC (%) | 69 | 71 | 61 | 73 | 71 | 69 | 92 | 75 |
| TLC (% pred.) | 123% | 94% | 117% | 112% | 100% | 112% | NA | NA |
| FRC (% pred.) | 128% | 98% | 139% | 112% | 112% | 137% | NA | NA |
| FEV_1_ (% pred.) | 124% | 90% | 98% | 112% | 97% | 106% | 104% | 133% |
| VC (% pred.) | 135% | 100% | 127% | 131% | 105% | 118% | 98% | 150% |
| RV (% pred.) | 117% | 98% | 118% | 101% | 108% | 120% | NA | NA |
| FEV_1_/VC (% pred.) | 87% | 93% | 79% | 94% | 94% | 92% | 109% | 96% |

Abbreviations: *V*_T_ tidal volume; *T*_bc_ time of breath cycle; *V*_e_ minute volume ventilation rate; DF_meas_ measured total deposited fraction; *R*_0_ zero seconds recovery and *r*_AiDA_ airspace size derived from AiDA measurements at TLC and *R*_0,1/2_ and *r*_AiDA,1/2_ are from AiDA measurements at half inflation. R_5_ and R_20_ respiratory resistance at 5 and 20 Hz from oscillometry, respectively; TLC total lung capacity; FRC functional residual capacity; FEV_1_ forced expiratory volume in 1 s; RV residual volume; VC vital capacity.

**Reference**

Quanjer PH, Tammeling GJ, Cotes JE, Pedersen OF, Peslin R, Yernault JC. Lung volumes and forced ventilatory flows. Report working party standardization of lung function tests, european community for steel and coal. Official statement of the european respiratory society. Eur Respir J Suppl. 1993;16:5–40.

Additional file 2

**Statistical analysis**

**Table A1.** Multiple-linear regression for DF_meas_ with VC, *R*_0_, *r*_AiDA.1/2_ as independent variables.

|  | Coefficient | Standard error | p |
| --- | --- | --- | --- |
| VC | 0.0110 | 0.018 | 0.543 |
| *R*_0_ | -0.0616 | 0.078 | 0.439 |
| *r*_AiDA,1/2_ | -0.0012 | 0.000 | 0.006 |
| Ln(*T*_bc_) | 0.1881 | 0.097 | 0.070 |
| Ln(*V*_T_) | 0.0914 | 0.108 | 0.410 |

**Table A2** Relationships between all lung function measurements and background variables with each other assessed with Pearson correlations. Significance levels (p-values) are indicated with asterisks: p<0.001 (***), 0.001≤p<0.01 (**) and 0.01≤p<0.05 (*).

|  | TLC | RV | FRC | VC | FEV_1_ | FEV_1_/VC | R_5_ | R_20_ | X_5_ | Ax | F_res_. | D_LCO_ | KCO | V_inh_ | R_0_ | r_AiDA_ | V_inh,1/2_ | R_0,1/2_ | r_AiDA,1/2_ |
| --- | --- | --- | --- | --- | --- | --- | --- | --- | --- | --- | --- | --- | --- | --- | --- | --- | --- | --- | --- |
| Age | 0.56*** | 0.88*** | 0.66*** | 0.31 | -0.07 | -0.8*** | -0.03 | -0.16 | 0.56*** | -0.27 | -0.3 | 0.11 | -0.63*** | 0.41* | 0.44** | 0.37* | 0.33 | 0.36 | 0.68*** |
| Height | 0.86*** | 0.47** | 0.39* | 0.86*** | 0.76*** | -0.24 | -0.4* | -0.55*** | 0.4* | -0.04 | 0.15 | 0.73*** | -0.14 | 0.81*** | 0.18 | 0.55*** | 0.82*** | 0.25 | 0.37 |
| Weight | 0.71*** | 0.24 | 0.13 | 0.76*** | 0.81*** | -0.02 | -0.22 | -0.43** | 0.41* | -0.1 | 0.1 | 0.67*** | -0.03 | 0.78*** | 0.11 | 0.51** | 0.79*** | 0.22 | 0.35 |
| TLC | 1.0*** | 0.69*** | 0.72*** | 0.92*** | 0.69*** | -0.46** | -0.57*** | -0.7*** | 0.53** | -0.25 | -0.03 | 0.74*** | -0.27 | 0.79*** | 0.45** | 0.49** | 0.84*** | 0.53** | 0.45** |
| RV | 0.69*** | 1.0*** | 0.88*** | 0.35 | 0.01 | -0.74*** | -0.55*** | -0.57*** | 0.48** | -0.37* | -0.22 | 0.26 | -0.52** | 0.33 | 0.47** | 0.39* | 0.3 | 0.37* | 0.62*** |
| FRC | 0.72*** | 0.88*** | 1.0*** | 0.46** | 0.11 | -0.67*** | -0.7*** | -0.68*** | 0.48** | -0.36 | -0.24 | 0.37* | -0.4* | 0.26 | 0.65*** | 0.26 | 0.31 | 0.51** | 0.45** |
| VC | 0.92*** | 0.35 | 0.46** | 1.0*** | 0.86*** | -0.29 | -0.38* | -0.55*** | 0.42* | -0.03 | 0.09 | 0.82*** | -0.06 | 0.84*** | 0.33 | 0.42* | 0.93*** | 0.47** | 0.24 |
| FEV_1_ | 0.69*** | 0.01 | 0.11 | 0.86*** | 1.0*** | 0.21 | -0.31 | -0.5** | 0.21 | 0.1 | 0.27 | 0.75*** | 0.1 | 0.74*** | 0.09 | 0.3 | 0.82*** | 0.23 | -0.01 |
| FEV_1_/VC | -0.46** | -0.74*** | -0.67*** | -0.29 | 0.21 | 1.0*** | 0.03 | 0.04 | -0.4* | 0.28 | 0.37* | -0.09 | 0.42** | -0.25 | -0.35 | -0.18 | -0.21 | -0.33 | -0.45** |
| R_5_ | -0.57*** | -0.55*** | -0.7*** | -0.38* | -0.31 | 0.03 | 1.0*** | 0.9*** | -0.33 | 0.26 | 0.12 | -0.52** | -0.06 | -0.13 | -0.35 | -0.22 | -0.24 | -0.3 | -0.33 |
| R_20_ | -0.7*** | -0.57*** | -0.68*** | -0.55*** | -0.5** | 0.04 | 0.9*** | 1.0*** | -0.24 | 0.04 | -0.16 | -0.6*** | 0.03 | -0.35 | -0.35 | -0.28 | -0.42* | -0.27 | -0.35 |
| X_5_ | 0.53** | 0.48** | 0.48** | 0.42* | 0.21 | -0.4* | -0.33 | -0.24 | 1.0*** | -0.85*** | -0.72*** | 0.2 | -0.4* | 0.39* | 0.58*** | 0.35 | 0.44** | 0.51** | 0.56*** |
| Ax | -0.25 | -0.37* | -0.36 | -0.03 | 0.1 | 0.28 | 0.26 | 0.04 | -0.85*** | 1.0*** | 0.96*** | -0.02 | 0.26 | -0.08 | -0.39* | -0.29 | -0.15 | -0.49** | -0.45** |
| F_res_. | -0.03 | -0.22 | -0.24 | 0.09 | 0.27 | 0.37* | 0.12 | -0.16 | -0.72*** | 0.96*** | 1.0*** | 0.14 | 0.18 | 0.13 | -0.3 | -0.15 | 0.05 | -0.36 | -0.32 |
| D_LCO_ | 0.74*** | 0.26 | 0.37* | 0.82*** | 0.75*** | -0.09 | -0.52** | -0.6*** | 0.2 | -0.02 | 0.14 | 1.0*** | 0.42* | 0.64*** | 0.09 | 0.3 | 0.7*** | 0.28 | 0.07 |
| KCO | -0.27 | -0.52** | -0.4* | -0.06 | 0.1 | 0.42** | -0.06 | 0.03 | -0.4* | 0.26 | 0.18 | 0.42* | 1.0*** | -0.18 | -0.5** | -0.21 | -0.16 | -0.31 | -0.49** |
| V_inh_ | 0.79*** | 0.33 | 0.26 | 0.84*** | 0.74*** | -0.25 | -0.13 | -0.35 | 0.39* | -0.08 | 0.13 | 0.64*** | -0.18 | 1.0*** | 0.17 | 0.4* | 0.95*** | 0.32 | 0.36 |
| R_0_ | 0.45** | 0.47** | 0.65*** | 0.33 | 0.09 | -0.35 | -0.35 | -0.35 | 0.58*** | -0.39* | -0.3 | 0.09 | -0.5** | 0.17 | 1.0*** | 0.35 | 0.19 | 0.5** | 0.52** |
| r_AiDA_ | 0.49** | 0.39* | 0.26 | 0.42* | 0.3 | -0.18 | -0.22 | -0.28 | 0.35 | -0.29 | -0.15 | 0.3 | -0.21 | 0.4* | 0.35 | 1.0*** | 0.41* | 0.37 | 0.78*** |
| V_inh,1/2_ | 0.84*** | 0.3 | 0.31 | 0.93*** | 0.82*** | -0.21 | -0.24 | -0.42* | 0.44** | -0.15 | 0.05 | 0.7*** | -0.16 | 0.95*** | 0.19 | 0.41* | 1.0*** | 0.43* | 0.3 |
| R_0,1/2_ | 0.53** | 0.37* | 0.51** | 0.47** | 0.23 | -0.33 | -0.3 | -0.27 | 0.51** | -0.49** | -0.36 | 0.28 | -0.31 | 0.32 | 0.5** | 0.37 | 0.43* | 1.0*** | 0.3 |


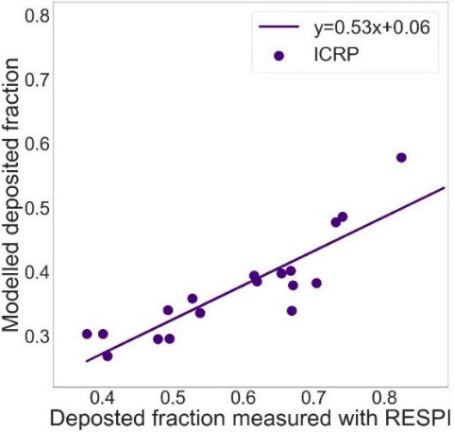

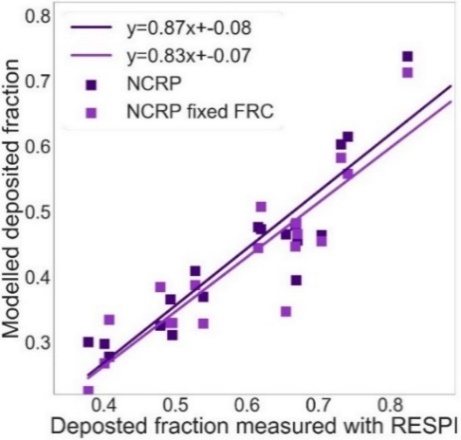


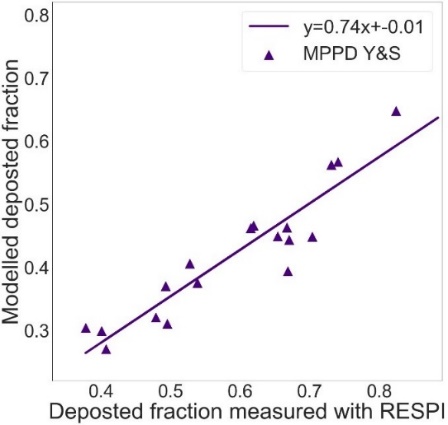

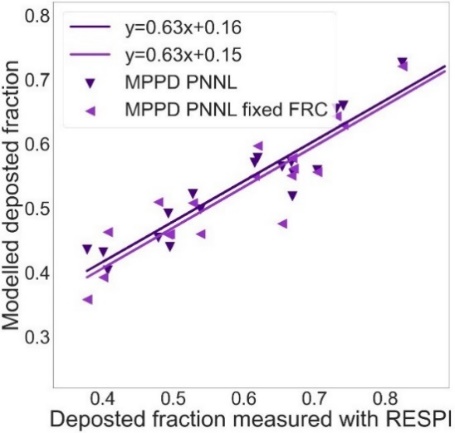

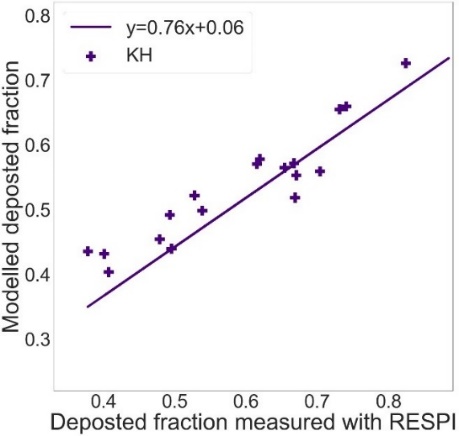


**Figure A1.** Modelled vs measured DF, for each model separately with linear regression line.

**Table A3** Pearson’s correlation coefficient, *r*, for the difference between modelled and measured DF with lung function indices measured. Significance levels (p-values) are indicated with asterisks: p<0.001 (***), 0.001≤p<0.01 (**) and 0.01≤p<0.05 (*).

|  | Diff_ICRP | Diff_NCRP | Diff_MPPD-Y&S | Diff_MPPD-PNNL | Diff_KH | Diff_NCRP_F_ | Diff_MPPD- PNNL_F_ |
| --- | --- | --- | --- | --- | --- | --- | --- |
| TLC | -0.36 | -0.07 | -0.32 | -0.38 | 0.46* | *0.5*** | *0.16* |
| RV | -0.01 | -0.16 | -0.08 | 0.03 | 0.6** | *0.58*** | *0.58*** |
| FRC | -0.1 | -0.35 | -0.25 | -0.11 | 0.54** | *0.51*** | *0.52*** |
| VC | -0.53** | -0.19 | -0.48** | -0.57** | 0.2 | *0.24* | *-0.16* |
| FEV_1_ | -0.42* | 0.01 | -0.32 | -0.47** | 0.01 | *0.07* | *-0.36* |
| FEV_1_/VC | 0.25 | 0.4* | 0.33 | 0.23 | -0.24 | *-0.19* | *-0.26* |
| R_5_ | -0.06 | 0.13 | 0.06 | -0.03 | -0.55** | *-0.54*** | *-0.47*** |
| R_20_ | 0.01 | 0.02 | 0.07 | 0.04 | -0.6*** | *-0.64**** | *-0.44** |
| DLCO | -0.4 | 0.03 | -0.26 | -0.38 | 0.28 | *0.35* | *-0.05* |
| KCO | -0.25 | 0.06 | -0.08 | -0.2 | -0.21 | *-0.13* | *-0.33* |
| *V*_tot_ | -0.28 | 0.27 | -0.09 | -0.28 | 0.25 | *0.27* | *-0.12* |
| *R*_0_ | 0.31 | -0.11 | 0.07 | 0.23 | 0.49** | *0.44** | *0.6*** |
| *r*_AiDA_ | 0.28 | 0.43* | 0.3 | 0.25 | 0.45* | *0.46** | *0.34* |
| *R*_0,1/2_ | -0.19 | -0.15 | -0.21 | -0.2 | 0.4 | *0.3* | *0.2* |
| *r*_AiDA,1/2_ | 0.58** | 0.49* | 0.51** | 0.55** | 0.7*** | *0.68**** | *0.74**** |
| *T*_bc_ | -0.45** | 0.2 | -0.24 | -0.46* | 0.02 | *0.2* | *-0.33* |
| *V*_T_ | -0.52** | 0.11 | -0.32 | -0.53** | 0.0 | *0.18* | *-0.35* |

**Table A4** Multiple-linear regression for difference between modelled and measured DF_diff_ with VC, *r*_AiDA,1/2_, *T*_bc_ and *V*_T_ as independent variables for all models that use FRC as input variable (Group A). All variables were centralized before regression.

| DIFF DF_mod_ - DF_meas_ | Variable | Coeff | Std error | P |
| --- | --- | --- | --- | --- |
| ICRP | VC | -0.0223 | 0.015 | 0.164 |
|  | *r*_AiDA1/2_ | 0.0011 | 0.000 | 0.010* |
|  | *T*_bc_ | 0.0077 | 0.015 | 0.618 |
|  | *V*_T_ | -0.1047 | 0.134 | 0.446 |
| NCRP | VC | -0.0319 | 0.014 | 0.038* |
|  | *r*_AiDA1/2_ | 0.0008 | 0.000 | 0.035* |
|  | *T*_bc_ | 0.0094 | 0.014 | 0.517 |
|  | *V*_T_ | 0.0197 | 0.125 | 0.876 |
| MPPD YS | VC | -0.0268 | 0.013 | 0.061 |
|  | *r*_AiDA1/2_ | 0.0007 | 0.000 | 0.033* |
|  | *T*_bc_ | 0.0041 | 0.013 | 0.759 |
|  | *V*_T_ | -0.0145 | 0.117 | 0.903 |
| MPPD PNNL | VC | -0.0236 | 0.013 | 0.088 |
|  | *r*_AiDA1/2_ | 0.0008 | 0.000 | 0.019* |
|  | *T*_bc_ | 0.0024 | 0.013 | 0.853 |
|  | *V*_T_ | -0.0478 | 0.114 | 0.681 |

Additional file 3

**Particle generation and system losses**

**Figure A1** APS data from measurements of the lung deposition of one subject. The measurement is divided into three periods, 5 minutes each. Upper panel shows the average spectra from APS 1 starting sampling from inhaled aerosol and lower panel from APS2, starting the sampling from the exhalation tank.

**Figure A2** Internal particle losses in the RESPI-system as measured by the APS at a constant flow through the system. Area marked as grey covers the difference found between the results in the loss calibration performed at 3 different occasion (over the course of time) and corresponds to the uncertainty in the internal particle losses.

Additional file 4

**Model results comparision**

**
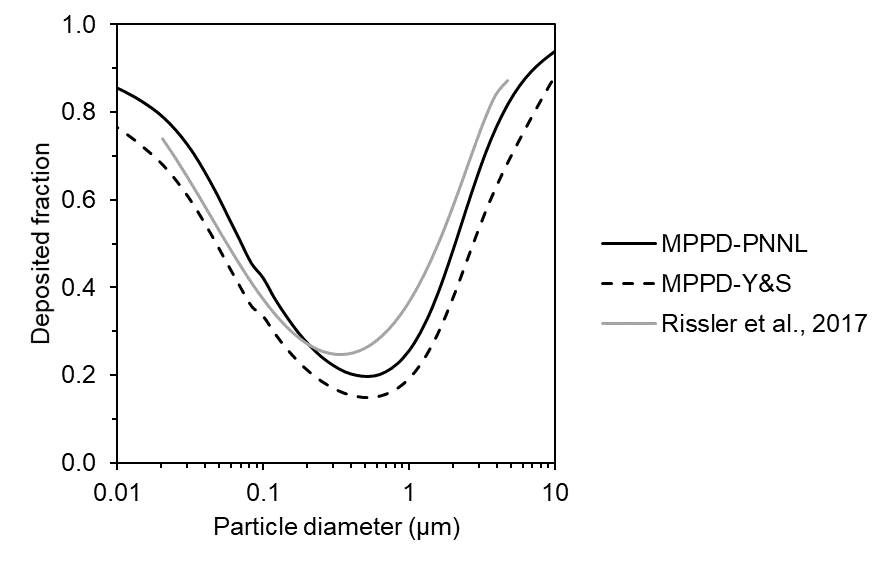
**

**Figure A1.** Total deposition of particles in the respiratory tract using MPPD-PNNL and MPPD-Y&S compared to the parametrization from Rissler et al. (2017). *V_T_* = 0.75 L, breathing frequency = 10 min^-1^, FRC = 3.4 L, extra-thoracic volume = 0.05 L. Note that in Figure 3 in the comparison to model in Rissler et al (2017), the MPPD modelling was performed using the symmetric lung model from Yeh and Schum (1980), whereas in this figure the modelling is done with the 5-lobe version.

**References**

Rissler J, Nicklasson H, Gudmundsson A, Wollmer P, Swietlicki E, Löndahl J. A set-up for respiratory tract deposition efficiency measurements (15–5000 nm) and first results for a group of children and adults. Aerosol Air Qual Res. 2017;17(5):1244–55.

Yeh HC, Schum GM. Models of human lung airways and their application to inhaled particle deposition. Bull Math Biol. 1980;42(3):461–80.
